# Zebrafish as a Model System to Screen Radiation Modifiers

**DOI:** 10.2174/138920207783406497

**Published:** 2007-09

**Authors:** Misun Hwang, Cha Yong, Luigi Moretti, Bo Lu

**Affiliations:** Department of Radiation Oncology, Vanderbilt Ingram Cancer Center, Vanderbilt University School of Medicine, Nashville, TN 37232, USA

## Abstract

Zebrafish (*Danio rerio*) is a bona fide vertebrate model system for understanding human diseases. It allows the transparent visualization of the effects of ionizing radiation and the convenient testing of potential radioprotectors with morpholino-modified oligonucleotides (MO) knockdown. Furthermore, various reverse and forward genetic methods are feasible to decipher novel genetic modifiers of radioprotection. Examined in the review are the radioprotective effects of the proposed radiomodifiers Nanoparticle DF-1 (C-Sixty, Inc., Houston, TX) and Amifostine (WR-2721, Ethyol), the DNA repair proteins Ku80 and ATM, as well as the transplanted hematopoietic stem cells in irradiated zebrafish. The presence of any of these sufficiently rescued the radiation-induced damages in zebrafish, while its absence resulted in mutagenic phenotypes as well as an elevation of time- and dose-dependent radiation-induced apoptosis. Radiosensitizers Flavopiridol and AG1478, both of which block progression into the radioresistant S phase of the cell cycle, have also been examined in zebrafish. Zebrafish has indeed become a favorite model system to test for radiation modifiers that can potentially be used for radiotherapeutic purposes in humans.

## I ZEBRAFISH AS AN IDEAL MODEL SYSTEM

Zebrafish (*Danio rerio*) has been envisioned as a popular vertebrate model system for studying radioprotection. The extensive forward genetic screens in the 1990s initiated the study of genes affecting zebrafish embryonic development [1,  2], and since then a large number of zebrafish mutants exhibiting rare human diseases have been obtained. Recent studies on zebrafish as a model system have provided important insight into various human diseases of oncogenic, neurodegenerative, hematopoietic, and cardiovascular origin [3-6]. Despite the evolutionary divergence nearly 450 million years ago, the human and zebrafish genome exhibit considerable homology with the conservation of key genes involved in development, signal transduction, cell cycle progression and proliferation, and cell differentiation [7-11]. The effects of ionizing radiation on the two organisms and the extent of phenotypic rescue by radioprotective substances are also similar. 

Most importantly, zebrafish offers far more practical benefits as a laboratory model system than any other organism. At low cost and with ease of care, large numbers of zebrafish can be maintained in a small space wherein frequent paired matings produce hundreds of embryos at a time. Its optical transparency of embryos allows the visualization of major organ systems during the rapid 3 months of embryonic development. Within 48 hours postfertilization (hpf), major organ systems such as the eyes, brain, heart, liver, muscles, bones, and the GI tract are evident. External embryogenesis also allows experimental manipulations to be conveniently carried out without parental sacrifice. Genetic manipulations in zebrafish, moreover, are relatively simple since the microinjection of morpholino-modified oligonucleotides complementary to the target gene or mRNA expressed from the target can be used to generate genetic knockouts [12,  13]. The morpholinos degrade after 4 days, however, making it difficult to assess the long-term effects of gene inhibition. Regardless, the 4 day time frame is adequate enough to evaluate the immediate effects of gene inactivation in zebrafish embryos and adults. 

The aforementioned benefits of using zebrafish as a vertebrate model system are only beginning to be appreciated, however, and the studies of radiation effects on zebrafish lag behind. Nonetheless, gamma-rays were shown to be an efficient mutagen in zebrafish [14,  15] that can generate a specific locus mutation rate of ~1:100. Subsequent gamma-ray mutagenesis studies produced interesting mutations affecting zebrafish embryogenesis [16-18]. A reverse genetic approach in the 1990s was then adopted to identify the affected genes from the isolated mutant phenotypes. Despite the significant efforts to establish a genetic map of the zebrafish genome [19,  20], far more needs to be done before the molecular mechanisms behind radiation-induced mutations are revealed and available for radiotherapeutic manipulations. 

## II RADIOPROTECTORS: DF-1 AND AMIFOSTINE

The success of cancer radiotherapy depends on the selective sparing of normal tissues. For decades, the enhancement of therapeutic index has thus been a central topic. As treatment strategies that can minimize radiation-induced toxicity, recent advances in radiation delivery technology such as Intensity-Modulated Radiotherapy (IMRT) [21,  22] and fractionation strategies [23] have been examined. More promising is the discovery of a large number of radioprotective substances [24], some of which selectively enter normal cells by unknown mechanisms. Notably, radioprotectors such as Amifostine and DF-1 have been shown to reverse the effects of total body irradiation (TBI) in animal models including zebrafish. Revealing their precise mechanism of radioprotection therefore has significant clinical implications.

### A DF-1

Nanoparticle DF-1 (C-Sixty, Inc., Houston, TX) is a water-soluble fullerene with antioxidant properties [25,  26]. Fullerenes refer to a family of molecules containing 20, 40, 60, 70, or 84 carbon atoms, and DF-1 is a C_60 _derivative (dendrofullerene) structurally modified to enhance water solubility and reduce toxicity. The unaltered Buckminster fullerene C_60 _has been demonstrated to exert toxic effects both *in vitro* and *in vivo*, such as in the aquatic largemouth bass [27]. Since fullerenes act as scavengers of reactive oxygen species (ROS) including hydrogen peroxide, hydroxyl radicals, and superoxide [28], it is thought that the antioxidant properties of DF-1 enables and accounts for its radioprotective role. If not blocked, the generated ROS can induce DNA damage and carcinogenesis [29,  30]. 

#### i Radioprotection in Zebrafish

A recent study by Daroczi and colleagues shows that DF-1 alleviates toxicity associated with irradiation of zebrafish embryos *in vivo *without exerting adverse effects on normal zebrafish morphology or viability throughout the concentration range tested (1-1,000 micromol/L). The group exposed zebrafish embryos to DF-1 at 24 hpf and monitored radiation effects up to day 6. The developmental stage of 24 hpf was preferably chosen since organ formation during this time is thought to be particularly sensitive to external radiation. Ionizing radiation ranging from 10 to 40 Gy caused time- and dose-dependent perturbations and lethality. Adverse radiation effects on the surviving zebrafish included defective midline development resulting in dorsal curvature of the body axis (“curly-up”) or *cup* [31], neurotoxicity, and impaired excretory function. The administration of DF-1 was able to reverse all of these side effects, the most noticeable by appearance being the reduced incidence of the *cup* phenotype. Edema due to impaired renal function is a well documented radiation side effect in mammals [32], and neurotoxicity is also a well known consequence of the central nervous system radiotherapy. The ability of DF-1 to diminish neurotoxicity was associated with the reduction of radiation-induced nerve cell damage and ROS production. The amount of stained neuromasts significantly decreased upon 80 Gy exposure at 50 days postfertilization (dpf), but such effect was inhibited by DF-1 pretreatment. The marked reduction of radiation-induced ROS was similarly measured using a fluorescent dye. The efficiency of DF-1 as a radioprotector depended upon its dose and time of application, however. The maximal radioprotective activity was exhibited at the concentration of 100μmol/L and when given within 3 hours before or up to 15 minutes after radiation (20Gy) exposure. When exposed to DF-1 30 minutes after ionizing radiation, zebrafish embryos were not protected from the harmful effects of radiation. Having ascertained the radioprotective effects of DF-1 in zebrafish, the next step would be to examine the molecular workings behind its dose- and time-dependent action.

### B Amifostine (WR-2721, Ethyol)

#### i Pharmacology

Nearly 50 years have passed since the discovery of thiol-containing amino acid cysteine as having remarkable radioprotective potentials [33]. The extensive testing of more than 4,000 sulfhydryl-containing substances then followed at the Walter Reed Army Institute of Research, ultimately yielding only one radioprotector amifostine as having acceptable toxicity [34]. Amifostine differs from DF-1 in that it possesses the ability to selectively protect normal tissues not only from irradiation but also from chemotherapy [35,  36]. Several studies have demonstrated its selective sparing of normal tissue when administered with several antineoplastic drugs, including cisplatin, carboplatin, carmustine, cyclophosphamide, doxorubicin, fluorouracil, melphalan, mitomycin, mechlorethamine, and paclitaxel [37-44]. In 1996, the Food and Drug Administration (FDA) approved the use of amifostine as a cytoprotective agent in cisplatin-based chemotherapy against ovarian cancer. Recently, amifostine has been approved for use in head and neck cancer to prevent radiation-induced xerostomia [45,  46]. It may also help ameliorate mucosal reactions that function as a limiting factor in accelerated fractionation or chemoradiation treatments. Many clinical trials are thus being carried out to test its potential use as a clinical radioprotector in broader terms [21,  47-51]. Clinical application of amifostine, however, is currently limited by its harmful side effects including hypotension, pruritus, and flushing [52]. Moreover, not one study so far has had the sufficient statistical power to conclude that amifostine does not reduce antitumor efficacy. 

Much attention has been given to the pharmacologic properties of amifostine due to its unique ability of conferring radioprotection only to normal cells. With a half-life of 1 to 3 minutes in plasma, amifostine is rapidly dephosphorylized to its active metabolite WR-1065 after intravenous administration [53]. Dephosphorylation is believed to be caused by spontaneous nonenzymatic hydrolysis or by an enzymatic catalysis involving alkaline phosphatase under the optimal pH of 8 or 9 [54]. Radiolabeled amifostine has revealed that its active metabolite WR-1065 accumulates in most normal tissues shortly upon injection into mice [54,  55], but preferentially more in certain tissues. Whereas extensive uptake was seen in salivary glands, kidneys, and intestinal mucosa, a markedly lower uptake was exhibited in tumor tissues [53-55]. The elimination rate of WR-1065 similarly varies considerably between tissues. The drug concentration in lung and skin decline rapidly during the first 30 minutes but remain elevated in salivary glands for up to 3 hours [55]. 

Several explanations have been offered to explain for such selectivity of tissues. The hydrophilic characteristics of amifostine make it difficult to cross cell membranes [36] or the blood-brain barrier [56,  57]. Despite the blood-brain barrier, amifostine has been shown neuroprotective effects when co-administered with chemotherapeutic agents [58,  59]. In contrast to normal tissues that can uptake amifostine by active transport, tumor cells absorb the agent *via *passive diffusion [54]. Given the unfavorable charge difference between amifostine and tumor cell membrane, passive diffusion may account for the significantly less uptake of the drug by tumor cells. Additionally, the differences between normal and tumor tissue perfusion, pH, hypoxia, and alkaline phosphatase activity have been regarded as potential selectivity determining factors [60-62]. 

Both oxygen-dependent and oxygen-independent pathways may be involved in the generation of radioprotection by amifostine. Substantial evidence suggests that oxygen concentration differences, rather than the tissue-dependent amifostine concentrations, may be of greater importance in conferring radioprotection [60,  62,  63]. In fact, WR-1065 and, to a lesser extent, its intracellular disulfide metabolic product WR-33278 act as free radical scavengers in protecting cellular damage [64,  65]. Independently of oxygen, it was shown that thiols may aid in chemical repair *via *hydrogen donation [66] and in the induction of DNA packaging which decreases the accessibility of radiolytic attack sites [64]. Furthermore, WR-33278 exhibits structural similarities to naturally occurring polyamines [67] known to influence DNA synthesis, DNA repair, gene expression, and cell cycle progression [67-69]. These characteristics of WR-33278 may explain the antimutagenic effect of amifostine even when administered up to 3 hours after irradiation [70].

#### ii Radioprotection in Zebrafish

Geiger *et al. *demonstrated in a zebrafish model that amifostine can ameliorate the toxic effects ionizing radiation [71,  72]. Embryos were exposed to varying degrees of gamma-radiation (5, 10, or 20 Gy) at sequential times post fertilization for viability and morphology assessments. Surviving embryos were markedly malformed, with shortened and curved bodies, defects in the head and eyes, pericardial edema, along with the inhibition of yolk sac resorption [72,  73]. Such radiation-induced malformations show similarity to that noted in mammals. Inadvertent or therapeutic exposure to radiation in humans reportedly results in cataract formation, retinal degeneration/atrophy, blindness, and microcephaly [74-80]. Of the many IR complications seen in the zebrafish study by Geiger and colleagues, the brain and eyes were markedly affected. The volume of the tectum of the brain decreased dramatically and the organization of retinal cellular layers was noticeably perturbed.

Not only was amifostine able to rescue the above phenotypes, it also blocked the caspase activation in the brain and eyes as detected through terminal deoxyribonucleotidyl transferase-mediated dUTP nick end labeling (TUNEL) assays. Previously shown with the TUNEL assay was the presence of apoptosis in the irradiated zebrafish hindbrain and the peripheral nervous system [73]. The level of apoptosis upon irradiation was seen through an *in vitro* assay detecting the bioluminescent imaging of caspase activity in zebrafish embryos. Treatment with amifostine or pancaspase inhibitor Z-VAD-fmk displayed a significant increase in light emission than without the treatment, signifying the presence of more apoptosis-resistant embryos. Of the two caspase levels measured, substantially more caspase 9 activation was noted than that of caspase 8. Interestingly, the amount of caspase activation at 20 Gy was not twice that of 10 Gy suggesting that the extent of caspase activation may not be proportional to the radiation dose beyond an “optimal” radiation dose range. To better account for the conditions under which amifostine enhanced the survival rate of irradiated zebrafish embryos, the group coined and defined the radioprotection ratio as the proportion of surviving embryos under amifostine and a given dose of IR treatment divided by the proportion surviving the same dose of IR in the absence of amifostine. Embryos were thus irradiated at 2, 4, 6, 8, or 24 hpf with 5, 10, 20 Gy and assessed every 24 hours until 144 hpf. The result showed that the radioprotective effects of amifostine depend on the age of the embryo at the time of irradiation as well as the dose delivered. At all doses of radiation, the greatest radioprotection was conferred to the younger embryos irradiated up to 8 hpf. 

Previous reports point out the correlation between embryonic age and radiosensitivity, a link that may provide an explanation to the variant radioprotection ratios based on the different times of amifostine and radiation administration. Many studies have also found increased radiosensitivity at earlier points of embryonic development. Gastrulation during zebrafish embryonic development refers to the first definable point at which organ development begins and generally occurs around 5 to 7 hpf [81]. It has been shown that the susceptibility of the embryos to the lethal effects of radiation diminishes after this point, bolstering the hypothesis that the times at or before gastrulation are particularly radiosensitive [15]. Exposure to IR during the sensitive ‘critical periods’ of organ-specific formation or morphogenesis has been demonstrated to cause tissue-specific malformation [82,  83]. Early reports with zebrafish mutagenesis similarly found enhanced radiosensitivity before the midblastula transition (MBT) as compared with later developmental stages [15]. However, Walker and colleagues did not score the effects of different radiation doses nor categorize the severity of mutations. To account for the time-dependency of radiosensitivity during embryonic development, McAleer *et al. *hypothesized that the noted nonspecific manifestations of irradiation mutagenesis during early embryonic development [2] may be due to the absence of repair proteins before MBT, before which maternal proteins present in yolk govern all processes. In other words, the reversal of radiation-incurred DNA damage seems to require the transcription of necessary repair genes by the embryo proper after MBT [84]. 

Evidences suggest that self-elimination, rather than damage reparation, may be the favored adaptive response to radiation during IR-sensitive periods such as the organogenic stage. Little or no radiation-induced cancer risk at this time point denotes a potential mechanism where the self-elimination pathway is chosen over the DNA damage repair pathway [85,  86]. Such self-elimination is now known to occur by the p53-dependent apoptosis pathway [87-89]. On the other hand, exposure to the low-dose IR during later developmental stages in rodent models [86,  90] and human fetus [91-94] develop chromosomal aberrations or mutations leading to cancer. It is important to note that most assays testing the effects of IR on zebrafish embryos are conducted just few days after postfertilization and therefore fails to accurately measure long-term consequences. With increased follow-up time, radiation may lead to alterations of behavior or responsiveness to the environment that can induce not an immediate death but a gradual lethal failure to thrive such as with impaired feeding capability [74,  76,  79,  80]. If advanced technology permits, radiotherapy may artificially induce DNA repair to reverse radiation damages. Given the harmful and sometimes lethal long-term effects of IR, however, it remains uncertain that damage repair is a preferred survival mechanism than self-elimination. 

### C DNA Repair Proteins: Ku80 and ATM 

Embryogenesis is a particularly radiosensitive stage of the vertebrate life cycle since the rapid cell division renders cells more vulnerable to the mistakes made by the error-prone DNA repair mechanisms. Of the many IR-induced DNA lesions in the nucleus, double-stranded breaks (DSBs) appear to be the most predominant mutagenic lesion [reviewed in [95]]. Upon DSB formation, irradiated cells enter the DNA damage-dependent cell cycle arrest in an attempt to reestablish chromosome integrity by fixing the lesion [96-99]. The DSB repair may occur either by nonhomologous end joining (NHEJ) or by homologous recombination [reviewed in [100, 101]]. If the repair is not properly carried out, a permanent cell cycle arrest, death, or oncogenesis may result [102-105]. Depending on the extent of DNA damage, however, a cell may resort to self-elimination by apoptosis instead [reviewed in [106]]. To date, the radioprotective function of only a few DNA repair proteins including the Ku proteins (Ku80 and Ku70) and ATM have been examined in irradiated zebrafish embryos.

#### i Ku80

The Ku protein, composed of Ku70 and Ku80 subunits, facilitates the initial DNA recognition step of NHEJ and is well studied in mammalian systems [107]. Several studies have cloned the zebrafish orthologue of human Ku80 involved in NHEF pathway of DSB repair [108-110] and confirmed its role in the radiation-triggered DNA damage response in zebrafish [73]. The Ku protein repair pathway requires the presence of other proteins other than Ku70 and Ku80: LIG4 and XRCC4 encoding two subunits of a DNA ligase and PRKDC encoding the DNA-dependent protein kinases catalytic subunit. Knocking out any of these components in mice results in severe radiosensitivity [reviewed in [100, 111]].

Bladen and colleagues demonstrated that the expression of Ku80 in irradiated zebrafish embryos contribute to radioresistance by promoting damage repair rather than apoptosis. The zygotic transcription of Ku80 mRNA, encoded by the XRCC5 gene, is followed by its accumulation in specific tissue domains primarily consisting of rapidly proliferating cells, including organ-specific progenitor cells. Such tissue populations include the proliferative zones of the retina and central nervous system, specifically the retinal ganglion cells in the developing retina and the ventricular surface of the brain [112]. Moreover, the zygotic expression of Ku80 mRNA significantly increased during gastrulation (6 hpf). The Ku80 function does not appear to be essential during embryonic development in the absence of genotypic stress, for zebrafish embryos with reduced Ku80 activity develop normally. However, when the Bladen group suppressed the Ku80 expression using antisense morpholino oligonucleotides and subjected zebrafish embryos to low dose irradiation (50 cGy) during gastrulation, apoptosis was markedly upregulated throughout the developing CNS. The apoptosis exhibited was shown to be p53-dependent, indicating its activation to be downstream of unrepaired DNA damage. The presence of DSB repair genes during early zebrafish developing therefore seems to play a critical role in shaping the balance between cell survival and apoptosis upon radiation stress. 

Interesting to note is the radiation dose- and time-dependency of apoptosis initiation after radiation treatment. Bladen *et al. *revealed the existence of a threshold radiation dose below which cell death does not occur. Zebrafish embryos exhibited the absence of ectopic apoptotic cell death at 15 cGy as compared to the substantial amount at only a 3-fold higher radiation dose. This result has limitations, however, in choosing the endpoint for TUNEL analysis to be 24 hpf after which the onset of embryonic pigmentation obscured the signal. In addition, the ability to undergo apoptosis has been shown to take place at ~7 hpf in zebrafish embryos [84]. Other studies have shown that IR-induced apoptosis initiation begins at later developmental time points such as the gastrulation stage [113-115]. Recently, Ku70 (XRCC6) was shown to play a similar radioprotective role as Ku80 [116] and possesses the unique function of inhibiting the Bax-mediated apoptosis [117-119]. Further examining the ways in which Ku70 interact with the apoptosis and repair pathway components may reveal the mechanism used to selectively choose either repair or self-elimination after irradiation. 

#### ii ATM

The ataxia-telangiectasia mutated (ATM) encodes a nuclear serine/threonine kinase [120, 121] which takes on a key role in early response to DNA damage incurred by irradiation [122-124]. Radiation activates ATM by triggering the dissociation of the inactive, dimeric ATM complex and the autophosphorylation of Ser^1981^. The stimulated ATM then localizes to ionizing radiation-induced foci (IRIF) to catalyze the phosphorylation of many effector proteins regulating DNA repair, cell cycle arrest, apoptosis, and transcription [96, 105]. The ATM substrates include the tumor suppressor p53, NBS1, H2AX, breast cancer susceptibility gene 1 (BRCA1), 53BP1, checkpoint kinase 2, 53BP1, Smc1, FANC2, H2AX, and Pin2/TRF1 [96, 122, 123, 125-131]. Mutation of the ATM gene causes ataxia-telangiectasia (A-T), a rare autosomal recessive disorder (affecting approximately 1 of 40,000 to 1 of 100,000) characterized by neurodegeneration, immunodeficiency, growth retardation, cancer predisposition and premature aging, as well as hypersensitivity to ionizing radiation [132, 133].

Various animal models have been utilized to study the radioprotective effects of ATM especially in the CNS. Several laboratories have generated ATM-knockout mice containing specific germline inactivation of the ATM gene [134, 135]. These mice display similar phenotypes as that of A-T patients even at cellular levels [136]. Common to all vertebrate models is the absolute requirement of ATM in the normal CNS development and in the protection of developing CNS against radiation damages. During gastrulation and early neurulation, the ATM expression is particularly elevated in the nervous system [120, 137]. ATM expression primarily in the CNS has been noted in Xenopus embryo [138] as well as in adult mouse and humans [139, 140]. Previously documented is the crucial role of ATM in eliminating neural cells with DNA damage [139, 141-143] through the apoptotic pathway [135].

Zebrafish ATM (zATM) and human ATM (hATM) share a high level of homology as revealed through the PCR-based sequence analysis. Notably, the putative FAT phosphoinositide 3-kinase (PI3K)-like and FATC domains common to both zATM and hATM function in regulating the ATM kinase activity [144] and represent the most highly conserved regions with 64-94% amino acid homology. Meanwhile, approximately 50% amino acid homology is exhibited outside these domains. In addition, dominant-negative mutation sites of hATM as well as a number of identified mutation sites in A-T patients are conserved in zATM [145-148]. Studying the effects of ATM expression during zebrafish development thus has important clinical implications in humans.

In the study by Imamura *et al.*, the zATM knockout zebrafish model exhibited a heightened radiosensitivity upon irradiation. By in situ hybridization, it was shown that the ubiquitous zATM mRNA dramatically increased at the 18-somite stage, then localized specifically in the eye, brain, and tail by 24 hpf and at later stages [149, 150]. Interestingly, the zATM mRNA present since 0.75 hpf (two-cell stage) were lost by 10 hpf (one-somite stage) but redetected at 19 hpf (20-somite stage). To examine the radioprotective effects of zATM, the group blocked the PI3K-like domain of zATM *via *splice-blocking antisense-morpholino oligonucleotides and subjected the zATM-deficient embryos to 8 Gy of radiation at 6 hpf. MO-based knockdown was highly penetrant in the first two days during which critical processes of somitogenesis and organogenesis that can heighten the sensitivity to ionizing radiation occur. The resultant phenotypes of irradiated embryos included the absence of pigmentation, lack of yolk extension, and extreme ventral body curvature. Treating the embryos with greater concentrations of zATM MO significantly increased the mortality rate at 48 hpf and led to complete lethality by 72 hpf when combined with ionizing radiation. Under 8 Gy IR, 96.2% of the embryos injected with zATM MO exhibited the abnormal phenotype whereas only 3.5% of those uninjected was affected by radiation. Consistent with the previous finding that featured enhanced radiosensitivity in AMT-deficient mice embryos [151], this study suggests zATM to be a guardian of genotoxic stress. 

### D Radioprotection *via *Hematopoietic Cell Transplantation (HCT)

Pioneering studies by several laboratories have traced the fates of the BMT cells and showed that donor bone marrow contains rare cells generating clonal, macroscopic spleen colonies [152, 153], a fraction of which regenerated spleen colonies [154], and repopulating multilineage hematopoiesis [155]. These experiments gave rise to the concept of the hematopoietic stem cell (HSC). The HSC transplantation has not only been used to test transformation in mouse cancer cell models but also to test the radioprotective properties of hematopoietic stem cells in irradiated zebrafish. Minimum lethal dose (MLD) has been shown to ablate cells of blood-forming tissues and nearly all leukocyte subsets residing in the kidney within a week before death. The seminal murine studies further demonstrated the rescue of acute irradiation syndrome by bone marrow transplantation (BMT) [156, 157]. Moreover, recent mouse studies indicate that the transplantation of megakaryocyte or erythrocyte progenitor cells is sufficient for radioprotection following the MLD [158]. 

With these evidences in hand, Traver and colleagues hypothesized that irradiation-induced mortality is due to hematopoietic failure and attempted to rescue irradiated zebrafish embryos by transplanting hematopoietic cells. A minimum lethal dose (MLD) of 40 Gy led to the specific ablation of hematolymphoid cells and death by 14 days after irradiation. Having found previously that the adult zebrafish kidney contains long-term HSCs and its downstream progenitor cells, [159], the group transplanted whole kidney marrow (WKM) cells into the lethally irradiated zebrafish 2 days after irradiation. If the hematopoietic failure is the only lethal consequence of a 40-Gy dose, then HCT should arrest the plummeting survival rate of the irradiated zebrafish. Indeed, WKM transplantation into these zebrafish resulted in a 70% survival rate over 30 days compared to the controls which all died by 14 days after irradiation. The transplanted WKM cells carried transgenes yielding red fluorescent erythrocytes and green fluorescent leukocytes, and this allowed the visualization of donor-derived cells repopulating the recipient hematolymphoid tissues. Previous experiments replicate the results of current study in showing that HCT sufficiently radioprotects normal tissues from otherwise lethal doses of radiation [156, 160-165]. It was once thought that humoral factors produced from the transplanted hematopoietic tissues act as radioprotective elements [164]. 

Traver and colleagues also points out the role of temperature in affecting the degree of radioprotection. Nearly 40 Gy of IR was required for the MLD in zebrafish, contrasting with only 9 to 10 Gy needed for the same effect in mammals. It was previously shown that cold-blooded animals (i.e. zebrafish) are more radioresistant than warm-blooded animals [166]. Additional data further confirm the strong negative correlation between temperature and radioresistance. In mice, for example, the temporary reduction in ambient temperature to 0 - 0.5°C allows the radiation dose required for half of the animals to die over 30 days (LD50/30) to increase from 6.2 Gy to 18 Gy [167]. Histologic studies in both mammals and teleosts demonstrate that radiation damages are less severe at lower temperatures at the cellular level [168]. Additionally, the life span of erythrocytes has also been shown to increase upon temperature reduction [169]. Given that blood cell repopulation is crucial for survival following irradiation, the observed correlation between radioresistance and temperature may be due to both to metabolic and life span alteration of irradiated erythrocytes until a complete recovery of host hematopoiesis.

## III RADIOSENSITIZERS: AG1478 AND FLAVOPIRIDOL

### A AG1478

Zebrafish as a model system has also been of great use in screening for potential radiosensitizers. McAleer and colleagues documented both the effects of radioprotector amifostine and radiosensitizer AG1478, a tyrosine kinase inhibitor, introducing the concept of using zebrafish not only to screen antineoplastic agents [5], but also to test radiation modifiers [72]. AG1478 is an epidermal growth factor receptor (EGFR) tyrosine kinase inhibitor shown to radiosensitize human cells *in vitro* and in preclinical xenograft experiments [170]. In comparison to the control, zebrafish embryos pretreated with 2.5-5 μM AG1478 and ±4 Gy X-rays at 4 hpf displayed approximately a 20% reduction in the survival rate and a significantly enhanced teratogenicity as early as 72 hpf. Although the mechanism behind the radiosensitizing action of AG147 was not mentioned, the experiment strongly suggests zebrafish as a model system to be used in the testing of not only radioprotectors but also radiosensitizers.

### B Flavopiridol

McAleer and colleagues further tested the radiosensitizing effects of a cell cycle inhibitor termed flavopiridol [171]. Flavopiridol is a small-molecule semisynthetic flavonoid that functions to inhibit cyclin dependent kinases [172] and affect various signal transduction cascades [173, 174]. The abrogation of G1/S cell cycle check point following the inhibition of cyclin D1 blocks the progression of cells into S phase, the stage which supposedly exhibits radioresistance [175]. Hence, the cyclin D1 inhibition *via *flavopiridol should radiosensitize cells to the harmful effects of IR as compared to controls. The *in vivo* radiosensitizing effect of the flavonoid has been exhibited in a murine model system xenografted with human [176] and syngeneic mouse tumors [177, 178]. Additionally, flavopiridol has been reported to induce radiosensitization in various human cancers including esophageal [179], prostate [180], ovarian [178], colon, gastric [181], and leukemic [182] carcinomas. The clinical trials [183] showed discouraging results, however, due to the significant toxicity when administered as monotherapy [184] and when co-administered with chemotherapeutic agents [185, 186]. 

Flavopiridol has been shown to radiosensitize zebrafish to the same extent as with antisense oligomers to cyclin D1. McAleer *et al. *treated 1- to 4-cell stage zebrafish embryos with 500 nM flavopiridol, previously concluded as the biologically active concentration [183], and with 0.5 pmol antisense hydroxylprolyl-phosphono peptide nucleic acid (HypNA-pPNA) oligomers to test the radiosensitizing effects of reducing cyclin D1 expression. Both treatments induced almost a twofold increase in mortality after exposure to 40 Gy by 96 hpf and more severe morphologic changes as compared to the control. Most noticeably, exposure to 40 Gy resulted in 100% of control embryos with defective midline development labeled as the cup or “curly up” phenotype, whereas only 20 Gy was necessary to induce the same result in flavopiridol or oligomer-treated embryos. Whereas none of the treated embryos survived under the maximal radiation dose of 40 Gy, approximately half of the irradiated control embryos remained viable by 120 hpf (p = 0.026). Evident from these results is that the inhibition cyclin D1 appears sufficient enough to account for the radiosensitization in flavopiridol and (HypNA-pPNA) oligomer-treated zebrafish embryos.

## IV FUTURE DIRECTIONS

Zebrafish has indeed proven to be an ideal model organism whose radiation shielding mechanisms may aid in the identification of radioprotectors and radiosensitizers pivotal to radiotherapy. Zebrafish studies on radioprotectors such as amifostine cast a new hope in resolving the dilemma of collateral tumor protection in the context of radioprotective agents. Studies on the effects of Flavopiridol and AG1478 in zebrafish also foreshadow a potential future scenario of selectively radiosensitizing tumor cells, although much needs to be done before it comes to fruition. When combined advanced radiotherapies such as the Intensity-Modulated Radiation Therapy (IMRT), such chemical radiation modifiers will help enhance the selective targeting of tumor tissues in the future. Given the high homology of repair genes and the similar mutagenic phenotypes between zebrafish and mammals under genotoxic stress, the radioprotection mechanisms in zebrafish thus has far-reaching clinical implications.
